# Micronutrient Synergy in the Fight against Hepatocellular Carcinoma

**DOI:** 10.3390/cancers4020323

**Published:** 2012-03-23

**Authors:** M. Waheed Roomi, Nusrath W. Roomi, Tatiana Kalinovsky, Aleksandra Niedzwiecki, Matthias Rath

**Affiliations:** Dr. Rath Research Institute, 1260 Memorex Drive, Santa Clara, CA 95050, USA; E-Mails: w.roomi@drrath.com (M.W.R.); n.roomi@drrath.com (N.W.R.); t.kalinovsky@drrath.com (T.K.) m.rath@drrath.com (M.R.)

**Keywords:** hepatocellular carcinoma, HepG2, SK Hep-1, nutrient synergy, cell proliferation, MMPs, u-PA, TIMPs, matrigel invasion, apoptosis

## Abstract

The incidence of hepatocellular carcinoma (HCC), once thought to be a rare tumor in North America, has rapidly increased in recent years in the United States. Current treatment modalities to halt the progression of this disease are only marginally effective. The mainstay treatment is liver transplantation, which is often confronted with donor shortage. Invasion, metastasis and recurrence contribute to the high mortality rate of this disease. Matrix metalloproteinases (MMPs) that degrade the extracellular matrix (ECM) have been associated with the progression, invasion and metastasis of the disease. We have developed strategies to strengthen the ECM collagen and inhibit MMPs through micronutrients such as lysine, proline and ascorbic acid. Addition of epigallocatechin gallate or green tea extract to these micronutrients synergistically enhanced anti-carcinogenic activity in HepG2 cells. Addition of certain other micronutrients, such as *N*-acetylcysteine, selenium, copper and zinc (NM) synergistically enhanced the anticancer activity of the mixture in a model of hepatocellular carcinoma using HepG2 cells. *In vitro* studies using HepG2 demonstrated that NM was very effective in inhibiting cell proliferation (by MTT assay), MMPs secretion (by gelatinase zymography), cell invasion (through Matrigel) and induction of apoptosis (by live green caspase). In addition, NM was shown to down-regulate urokinase plasminogen activator (by fibrin zymography) and up-regulate tissue inhibitors of metalloproteinases (by reverse zymography) in another HCC cell line, SK-Hep-1. MMP-2 and MMP-9 activities were further modulated by phorbol 12-myristate 13-acetate (PMA) induction and inhibited by NM. In previous studies, NM inhibited Sk-Hep-1 xenografts in nude mice and also inhibited hepatic metastasis of B16FO melanoma cells. Our results suggest that NM is an excellent candidate for therapeutic use in the treatment HCC by inhibiting critical parameters in cancer development and progression, such as proliferation, invasion and metastasis, and by inducing apoptosis.

## 1. Introduction

Incidence of hepatocellular carcinoma (HCC) has dramatically increased worldwide in both sexes and all races in the past two decades [1]. HCC incidence tripled in the United States between 1975 and 2005, attributed to increased rates of hepatitis C virus infection [2]. Despite advances in its clinical study, the prognosis of hepatocellular carcinoma, which is diagnosed in over 700,000 people worldwide annually, remains dismal [2,3]. The most prevalent causes of death in patients with HCC include uncontrolled metastasis and recurrence. The progressive steps of metastasis include detachment of cancer cells from the primary tumor, disruption of the basement membrane, invasion into the surrounding stroma, cancer cell entry into and transport through the vascular or lymphatic system to distal sites such as the liver, lungs, and brain, and extravasation, tumor cell proliferation and angiogenesis at distal sites [4,5,6,7,8].

Thus, recent research has focused on exploring many molecular markers related to invasion, metastasis, recurrence and survival in HCC, such as: DNA ploidy, tumor cell proliferation, tumor suppressor and promoter genes, cell cycle controllers, proteinases that degrade extracellular matrix, adhesion molecules, angiogenic factors and metabolic genes [9]. Among these factors, the matrix metalloproteinases (MMPs) and the plasminogen activation system play crucial roles in cancer invasion and metastasis. Levels of MMP expression were found correlated to recurrence and reduced survival after HCC resection [10,11].

MMPs, a family of zinc and calcium dependent proteolytic enzymes, degrade connective tissue, among other substrates, such as basement membrane collagen, and have been associated with cancer metastasis and tumor angiogenesis. The gelatinases, especially MMP-9 (gelatinase B) and MMP-2 (gelatinase A), play a key role in degradation of collagen type IV and gelatin, two main components of the extracellular matrix (ECM). These gelatinases are expressed in hepatocellular carcinoma cells and are associated with progression and invasion of these tumors [10,12,13,14,15]. For example, Guo *et al*. noted positive correlation of MMP-9, MMP-2 and VEGF expression with recurrence of HCC [15].

Rath and Pauling [16] postulated that nutrients such as lysine and ascorbic acid could act as natural inhibitors of ECM proteolysis and, as such, have the potential to modulate tumor growth and expansion. These nutrients can exercise their anti-tumor effect through the inhibition of MMPs and by strengthening connective tissue surrounding cancer cells by influencing collagen synthesis. These two processes are essential for a tumor encapsulating effect. We have developed strategies to inhibit cancer development and its spread using naturally occurring nutrients such as lysine, proline, ascorbic acid and green tea extract plus other micronutrients (NM). This nutrient mixture has exhibited synergistic anticancer activity *in vivo* and *in vitro* in a number of cancer cell lines through inhibition of cancer cell growth, MMP secretion, invasion, metastasis, and angiogenesis [17,18,19].

We designed NM by defining critical physiological targets in cancer progression and metastasis, such as ECM integrity and MMP activity. ECM formation and structure is dependent upon adequate supplies of ascorbic acid and the amino acids lysine and proline, which insure proper synthesis and hydroxylation of collagen fibers. Manganese and copper are also essential cofactors in collagen formation. Lysine, a natural inhibitor of plasmin-induced proteolysis, plays an important role in ECM stability [16,20]. Green tea extract has been shown to modulate cancer cell growth, metastasis, angiogenesis, and other aspects of cancer progression [21,22,23,24,25]. *N*-acetylcysteine has been observed to inhibit MMP-9 activity [26] and invasive activities of tumor cells [27]. Selenium has been shown to interfere with MMP secretion and tumor invasion [28], as well as migration of endothelial cells through ECM [27]. In addition to addressing ECM properties, some nutrients are critical in inducing cancer cell death. A recent study confirmed that ascorbic acid inhibits cell division and growth through production of hydrogen peroxide [29]. Since arginine is a precursor of nitric oxide (NO), any deficiency of arginine can limit the production of NO, which has been shown to predominantly act as an inducer of apoptosis, as in breast cancer cells [30].

In a previous study on the effect of NM on hepatic metastasis in 10–12 week old athymic mice injected with 10^6^ murine B16FO melanoma cells, we found that mice provided a diet supplemented with NM 0.5% for two weeks showed not only significantly inhibition of tumor growth in the spleen (by 64%, *p* = 0.001) compared to mice fed the control diet, but also drastically reduced metastasis to the liver (by 55%, *p* = 0.006) [31].

In another study, we examined the effect of NM on human HCC cell line SK-Hep-1, *in vivo*, in athymic nude mice bearing SK-Hep-1 xenografts, and *in vitro*, evaluating viability, MMP secretion, invasion and induction of apoptosis [32]. We found that four weeks of NM (0.5%) supplementation of 4–6 week old male athymic nude mice after inoculation with 3 × 10^6^ SK-Hep-1 cells, profoundly inhibited the growth of SK-Hep-1 xenograft tumors compared to mice fed the normal murine diet. Mean tumor weight was inhibited by 42% (*p* = 0.09) with NM 0.5% dietary supplementation and tumor burden was inhibited by 36% (*p* = 0.005). *In vitro*, NM exhibited 33% inhibition of cell proliferation over the control at 500 and 1,000 µg/mL concentration. Zymography demonstrated secretion of MMP-2 and MMP-9, which was inhibited by NM in a dose dependent fashion, with virtual total inhibition at 1,000 µg/mL. Invasion through Matrigel was inhibited at 100, 500 and 1,000 µg/mL by 53%, 83% and 100%, respectively. NM induced slight apoptosis at 100 µg/mL, and profound apoptosis at 500 µg/mL and 1,000 µg/mL concentration [32].

Our main objective in the current series of studies was to compare the *in vitro* anti-cancer effects of epigallocatechin gallate (EGCG) alone, in combination with lysine, proline and ascorbic acid (LPA), and in combination with additional micronutrients, such as *N*-acetylcysteine, selenium, copper and zinc (NM) in a model of hepatocellular carcinoma using HepG2 cells. *In vitro* studies included analysis of cell proliferation (by MTT assay), MMPs secretion (by gelatinase zymography), and cell invasion (through Matrigel). HepG2 studies included the effect of NM on apoptosis (green caspase). In addition, we studied the effect of NM in another HCC cell line, SK-Hep-1, evaluating its efficacy on modulation of MMP-2 and -9, urokinase plasminogen activator (by fibrin zymography), and tissue inhibitors of metalloproteinases (by reverse zymography). HepG2 and SK-Hep-2 were selected for these studies as they are the standard cell lines used in hepatocellular carcinoma research.

## 2. Results and Discussion

### 2.1. Comparative Effect of LPA, EGCG and NM on HepG2 Cell Proliferation

**Figure 1:**
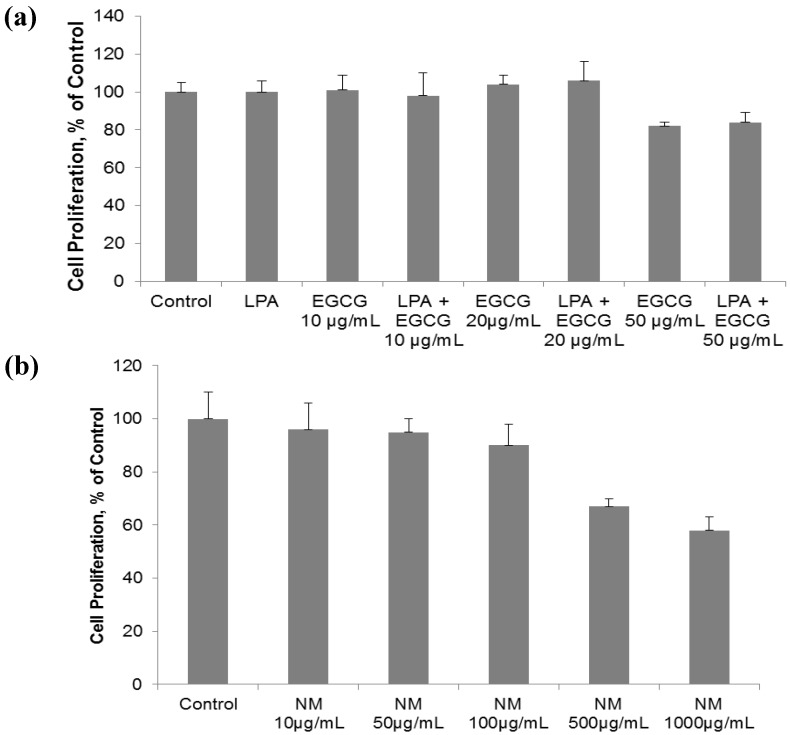
Comparative effect of EGCG, LPA and NM on HepG2 cell proliferation: MTT assay 24 h (**a**) Effect of EGCG with and without LPA on HepG2 cell proliferation; (**b**) Effect of NM on HepG2 cell proliferation.

Human hepatocarcinoma HepG2 cell proliferation was unaffected by EGCG with and without lysine, proline and ascorbic acid (LPA) at concentrations of 20 µg/mL and below. However, EGCG at 50 µg/mL, with and without LPA, inhibited cell proliferation by 26% and 28%, respectively, compared to the control (Figure 1a). When additional nutrients, designated as NM, were added to EGCG and LPA, inhibition of HepG2 cell proliferation was increased to 33% (*p* = 0.005) and 42% (*p* = 0.003) at NM 500 µg/mL and 1,000 µg/mL, respectively (Figure 1b). The equivalent of EGCG in NM 500 µg/mL is 38.3 µg/mL and in 1,000 µg/mL is 76.6 µg/mL. The concentration of LPA tested is equivalent to that found in NM 1,000 µg/mL. Thus, the combination of nutrients in NM acts synergistically to enhance the activity of EGCG and LPA beyond an additive effect.

### 2.2. Comparative Effect of LPA, EGCG and NM on HepG2 MMP Secretion

**Figure 2:**
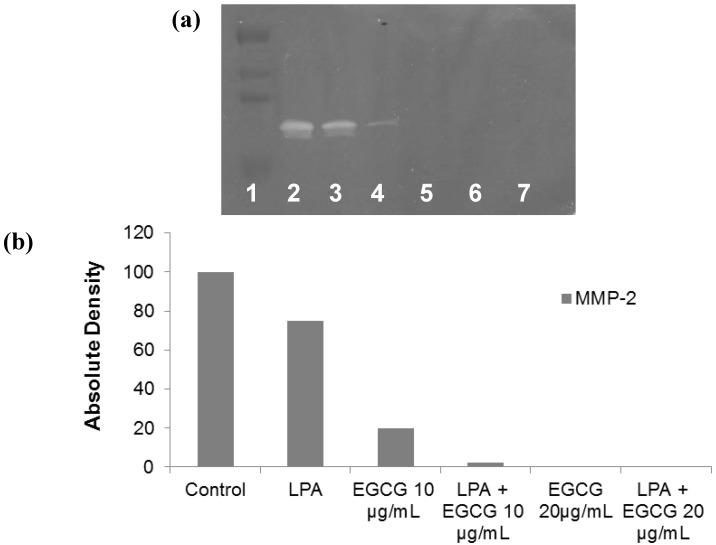
Comparative effect of EGCG with and without LPA on HepG2 MMP secretion. (**a**) Effect of EGCG with and without LPA on HepG2 MMP secretion: gelatinase zymogram. 1: Markers, 2: control, 3: LPA, 4: EGCG 10 µg/mL, 5: LPA + EGCG 10 µg/mL, 6: EGCG 20 µg/mL, 7: LPA + EGCG 20 µg/mL; (**b**) Effect of EGCG with and without LPA on HepG2 MMP secretion: densitometric analysis.

**Figure 3:**
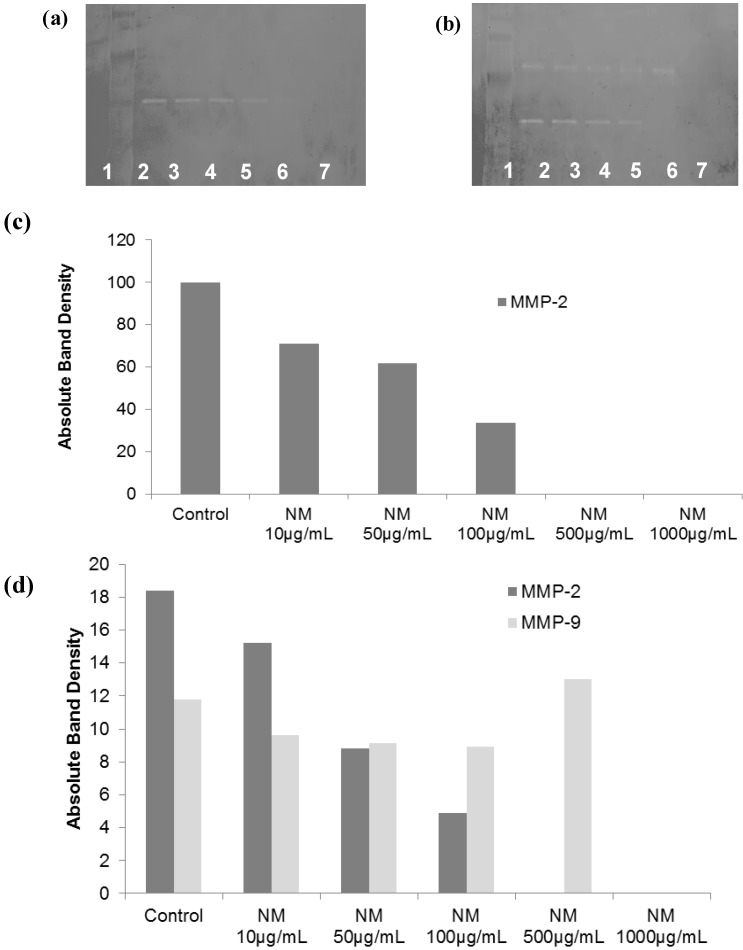
Effect of NM on HepG2 MMP secretion (**a**) Normal HepG2 MMP secretion: gelatinase zymogram; (**b**) PMA (100 ng/mL)-treated HepG2 MMP secretion: gelatinase zymogram. 1: Markers, 2: control, 3–7: NM 10, 50, 100, 500, 1,000 µg/mL; (**c**) densitometric analysis of normal HepG2 cell MMP secretion (d) densitometric analysis of PMA-treated HepG2 cell MMP secretion.

Gelatinase zymography demonstrated secretion of MMP-2 by untreated hepatocellular carcinoma HepG2. EGCG inhibited MMP-2 in a dose-dependent manner, which was enhanced with LPA; EGCG 10 μg/mL inhibited MMP-2 secretion by 80%, which was increased to 97.5% inhibition with addition of LPA to that concentration of EGCG (Figure 2). NM inhibited HepG2 MMP-2 secretion by 29% at a concentration of 10 μg/mL, by 66% at 100 μg/mL and completely blocked it at 500 μg/mL (linear trend: R^2^ = 0.962) compared to control (Figure 3). PMA (100 ng/mL) treatment of HepG2 resulted in induction of MMP-9 secretion, which was inhibited by NM by 23% at 50 μg/mL, and completely blocked at 1,000 μg/mL (linear trend: R^2^ = 0.328) compared to control (Figure 3). Gelatinase zymograms of HepG2 MMP expression are shown in Figure 2a for EGCG and LPA treatment and in Figure 3a for NM treatment. Densitometric analyses of HepG2 MMP expression are shown in Figure 2b for EGCG and LPA treatment and in Figure 3b for NM treatment.

### 2.3. Comparative Effect of EGCG, LPA and NM on HepG2 Matrigel Invasion

**Figure 4:**
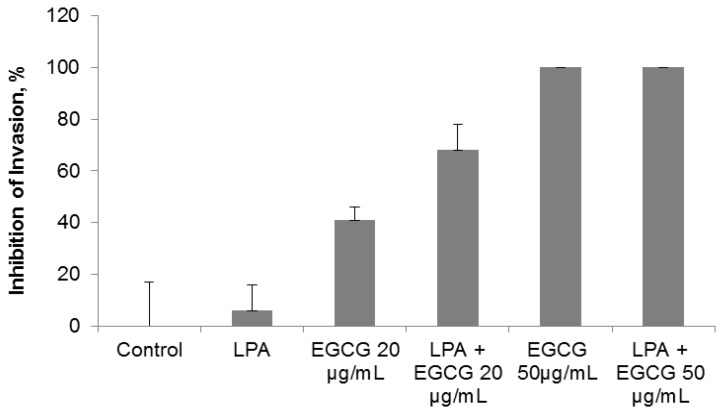
Comparative effect of EGCG with and without LPA on HepG2 cell invasion through Matrigel.

**Figure 5:**
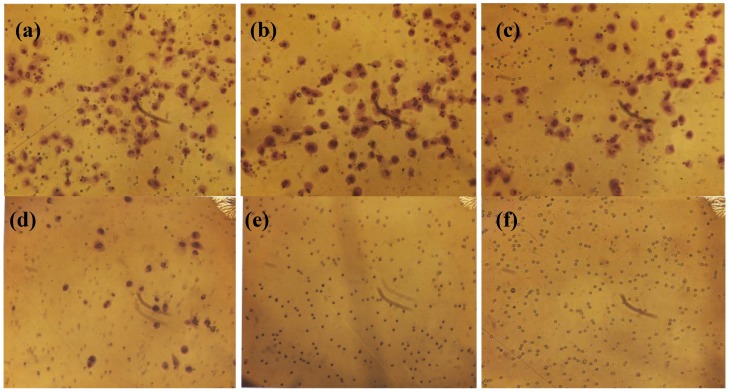
Comparative effect of EGCG with and without LPA on HepG2 cell invasion through Matrigel: photomicrographs (**a**) Control no LPA, (**b**) Control with LPA, (**c**) EGCG 20 µg/mL, (**d**) LPA + EGCG 20 µg/mL, (**e**) EGCG 50 µg/mL, (**f**) LPA + EGCG 50 µg/mL.

**Figure 6:**
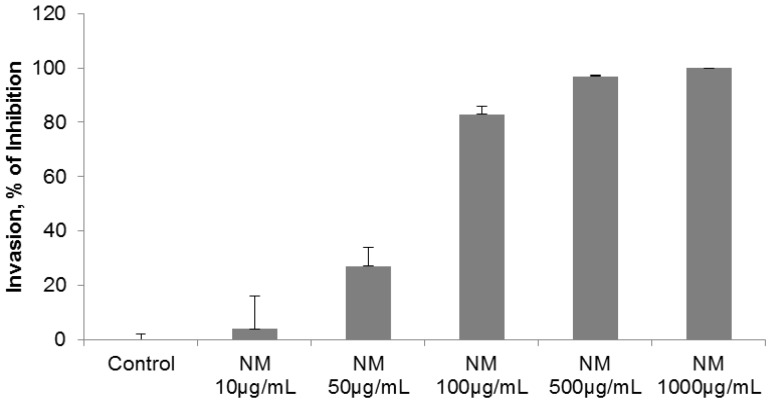
Effect of NM on HepG2 cell invasion through Matrigel.

**Figure 7:**
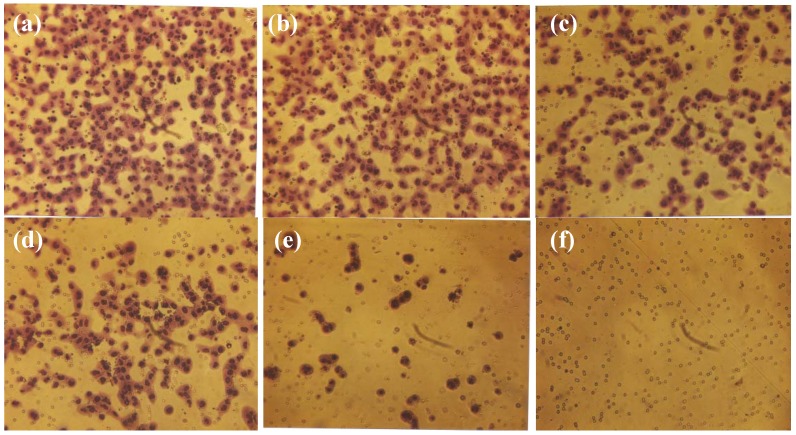
Effect of NM on HepG2 cell invasion through Matrigel: photomicrographs. (**a**) Control, (**b**) NM 10 µg/mL, (**c**) NM 50 µg/mL, (**d**) NM 100 µg/mL, (**e**) NM 500 µg/mL, (**f**) NM 1,000 µg/mL.

Individually, LPA and EGCG 20 µg/mL inhibited HepG2 invasion through Matrigel by 6% and 41%, respectively. However, when LPA acted in combination with EGCG 20 µg/mL, inhibition of invasion was enhanced to 68% (*p* = 0.004) over the control. As shown in Figure 4 and Figure 5, EGCG 50 µg/mL with and without LPA completely blocked HepG2 cell invasion through Matrigel. HepG2 cell invasion through Matrigel was significantly inhibited by NM: 27% (*p* = 0.003) at 50 µg/mL, 83% (*p* < 0.001) at 100 µg/mL, 97% (*p* < 0.0001) at 500 µg/mL and completely blocked at 1,000 µg/mL (linear trend R^2^ = 0.912). See Figure 6 and Figure 7. The equivalent of EGCG in NM 100 µg/mL is 15.3 µg/mL, in NM 500 µg/mL is 38.3 µg/mL and in NM 1,000 µg/mL is 76.6 µg/mL. To summarize, EGCG 20 µg/mL inhibited invasion by 41%. When LPA was added to EGCG 20 µg/mL, inhibition increased to 68%, and when other nutrients were added to this combination (NM), at a concentration of 100 µg/mL, (which is equivalent to a dose of 15 µg/mL EGCG), inhibition increased to 83%. Thus, NM exhibited superior potency in inhibiting HepG2 cell invasion through Matrigel compared to EGCG alone and to EGCG in combination with LPA at equivalent doses to that found in NM. These results suggest the importance of nutrients working in cooperation to more effectively treat complex biochemical pathways.

### 2.4. Effect of NM on HepG2 Apoptosis

**Figure 8:**
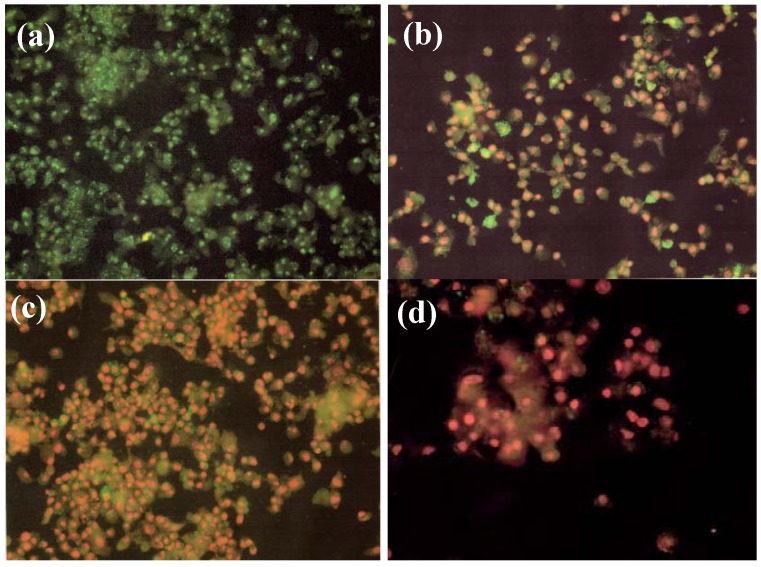
Effect of NM on HepG2 apoptosis: photomicrographs (**a**) Control, (**b**) NM 100 µg/mL, (**c**) NM 500 µg/mL, (**d**) NM 1,000 µg/mL.

**Figure 9:**
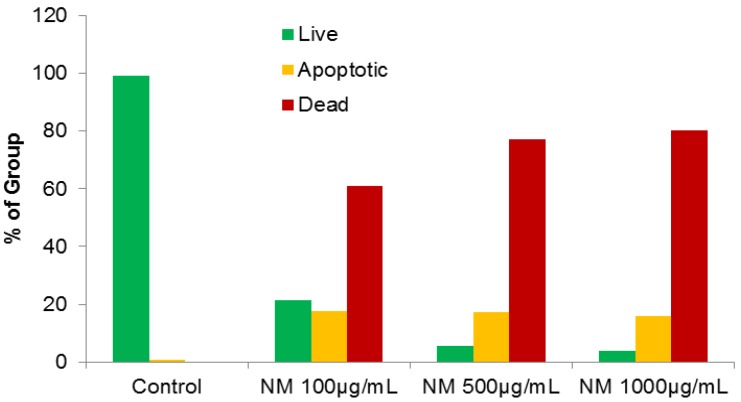
Effect of NM on HepG2 apoptosis: quantitative analysis.

Using the live green caspase kit, dose-dependent apoptosis of HepG2 cells was evident with NM challenge, as shown in Figures 8a–d. Approximately 79% of cells exposed to 100 µg/mL NM were apoptotic; the number of apoptotic cells increased significantly with increased NM concentration. Quantitative analysis of live, early and late apoptotic cells is shown in Figure 9. At 100 µg/mL NM, 21% of cells were viable, 18% in early apoptosis and 61% in late apoptosis and at 500 µg/mL NM 6% of cells were viable, 17% in early apoptosis, and 77% in late apoptosis. Virtually all cells exposed to 1,000 µg/mL NM were in apoptosis: 4% viable, 16% in early apoptosis and 80% in late apoptosis

### 2.5. Effect of NM on u-PA, MMPs and TIMPs Activities in SK-Hep-1 Cells

Two families of proteases, the matrix metalloproteinases and urokinase plasminogen activators (u-PA) are involved in tumor invasion and metastasis. Numerous clinical and experimental studies have demonstrated that elevated levels of u-PA and MMPs are associated with tumor growth, cancer progression, metastasis and shortened survival in patients [10,11,12,13,14,33,34]. Tumor cell invasion occurs secondary to degradation of the extracellular matrix, which is composed of collagen, proteoglycans, fibronectin, laminin and other glycoproteins [35,36,37]. The ECM acts as a barrier to block tumor growth and invasion of cancer cells. MMPs, a special family of over 20 zinc and calcium-dependent proteases, especially MMP-2 and MMP-9, play key roles in tumor cell invasion and metastasis due to their ability to degrade type IV collagen, a major component of the ECM [37,38,39]. Proteolytic activities of MMP-2 and MMP-9 are inhibited by specific inhibitors, tissue inhibitors of metalloproteinases. Thus, a critical determinant of net proteolytic degradation is the balance between levels of MMPs and TIMPs. The serine protease u-PA, a 55 kDa serine protease consisting of two disulfide bridges linked to polypeptides, is cleaved to the active chain (33 kDa) by various stimuli. The protease u-PA converts plasminogen to plasmin, which is capable of promoting tumor growth and angiogenesis, degrading the ECM and basement membrane and activating pro-MMPs [40].

#### 2.5.1. Effect of NM on u-PA activity in SK-Hep-1 Cells

**Figure 10:**
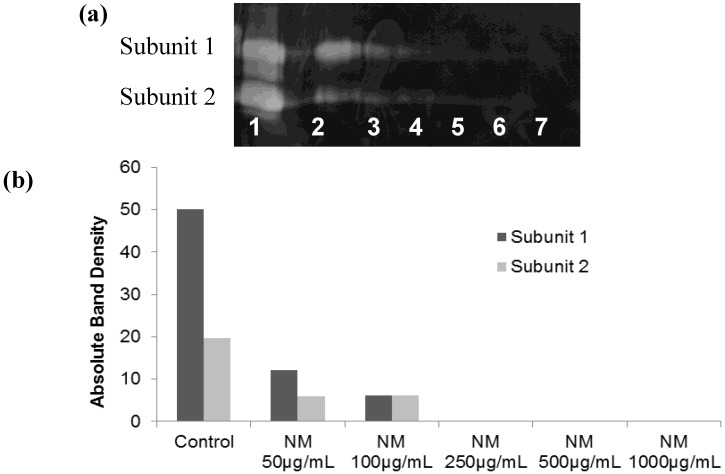
Effect of NM on SK-Hep-1 u-PA secretion (**a**) gelatinase zymogram; 1: Markers, 2: Control, 3–7: NM 10, 50, 100, 500, 1,000 µg/mL. (**b**) densitometric analysis.

Hepatocellular carcinoma SK-Hep-1 expressed u-PA, showing two bands corresponding to molecular weights 35 and 33 kD. SK-Hep-1 secretion of u-PA subunit 1 was decreased by 76% at 50 µg/mL, 88% at 100 µg/mL and was completely blocked at 250 µg/mL NM, compared to the control (linear trend: R^2^ = 0.639). Secretion of subunit 2 by SK-Hep-1 was inhibited by 69–70% at 50 µg/mL and 100 µg/mL, and completely blocked at 250 µg/mL NM, compared to the control (linear trend: R^2^ = 0.732. The fibrin zymogram of SK-Hep1 u-PA expression is shown in Figure 10a and densitometry analysis of u-PA expression in Figure 10b.

#### 2.5.2. Effect of NM on SK-Hep-1 Secretion of MMPs

**Figure 11:**
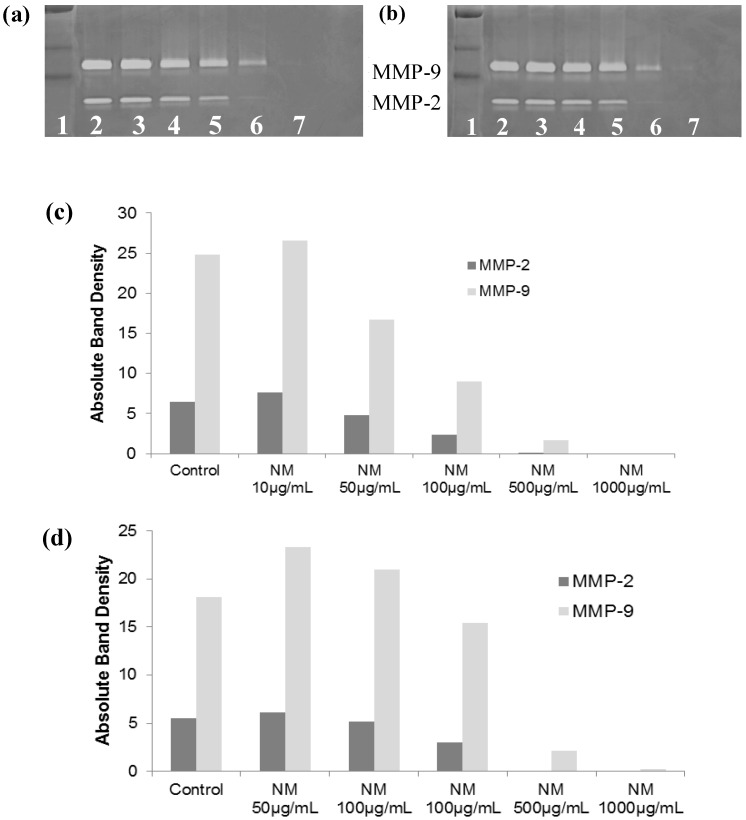
Effect of NM on SK-Hep-1 MMP-2 and -9 secretion: Gelatinase zymograms of (**a**) normal and (**b**) PMA-treated SK-Hep-1 cells; 1: Markers, 2: Control, 3–7: NM 10, 50, 100, 500, 1,000 µg/mL. Densitometry analysis of (**c**) normal and (**d**) PMA-treated SK-Hep-1 cells.

Gelatinase zymography demonstrated secretion of MMP-2 and MMP-9 by untreated (Figures 11a,b) and PMA (100 ng/mL)-treated (Figures 11c,d) hepatocellular carcinoma SK-Hep-1 cells. NM inhibited both MMPs in a dose-dependent manner. NM inhibited normal SK-Hep1 cell MMP-2 secretion by 25% at 50 µg/mL, 65% at 100 µg/mL, 98% at 500 µg/mL and totally blocked it at 1,000 µg/mL. MMP-9 secretion by normal SK-Hep-1 cells was inhibited by 33% at NM 50 µg/mL, 64% at 100 µg/mL, 99% at 500 µg/mL and was totally blocked at 1,000 µg/mL. PMA-treated SK-Hep-1 cells showed the same pattern of MMP-2 and 9 secretion and response to NM treatment.

#### 2.5.3. Effect of NM on TIMPs Activity in SK-Hep-1 Cells

**Figure 12:**
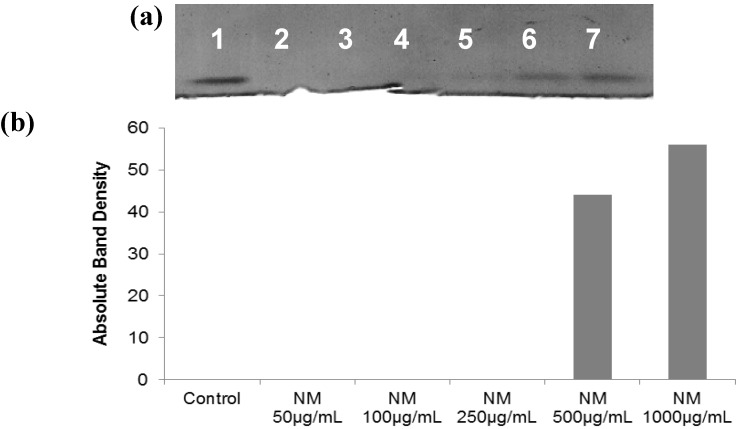
Effect of NM on SK-Hep-1 TIMPs secretion (**a**) gelatinase zymogram; 1: Markers, 2: Control, 3–7: NM 10, 50, 100, 500, 1,000 µg/mL. (**b**) densitometry analysis.

Activity of TIMPs was upregulated by NM in SK-Hep-1 cells (Figures 12a and b) in a dose-dependent manner. NM-treated SK-Hep-1 cancer cells demonstrated a significant increase (44%) in TIMP-2 activity at 500 μg/mL NM, which achieved a maximum increase of 56% at 1,000 μg/mL NM compared to no activity for the control (linear trend: R^2^ = 0.712).

Thus, NM demonstrated signiificant modulation of SK-Hep-1 invasive paramters by inibiting u-PA and MMP-2 and MMP-9 secretion and up regulating TIMPs expression.

## 3. Experimental Section

### 3.1. Cancer Cell Lines and Reagents

Human hepatoma cell lines HepG2 and SK-Hep-1 and recommended media were purchased from ATCC (American Type Culture Collection, Manassas, VA, USA). Penicillin, streptomycin, PMA, and fetal bovine serum (FBS) were obtained from Sigma (St. Louis, MO, USA). All other reagents used were of high purity and were obtained from Sigma, unless otherwise indicated.

### 3.2. Composition of the Nutrient Mixture

The nutrient mixture (NM) was composed of the following in the ratio indicated: Vitamin C (as ascorbic acid and as Mg, Ca, and palmitate ascorbate) 700 mg; L-lysine 1,000 mg; L-proline 750 mg; L-arginine 500 mg; *N*-acetylcysteine 200 mg; standardized green tea extract (derived from green tea leaves), was obtained from US Pharma Lab (Santa Clarita, CA, USA); the certificate of analysis indicated the following characteristics: total polyphenol 80%, catechins 60%, epigallocatechin gallate (EGCG) 35%, and caffeine 1.0%); 1,000 mg; selenium 30 µg; copper 2 mg; manganese 1 mg.

### 3.3. Cell Culture

Human hepatoma cell lines HepG2 and SK-Hep-1 were grown in MEM, supplemented with 10% fetal bovine serum, penicillin (100 U/mL) and streptomycin (100 mg/mL) in 24-well tissue culture plates (Costar, Cambridge, MA, USA). Cells were incubated with 1 mL of media at 37 °C in a tissue culture incubator equilibrated with 95% air and 5% CO_2_. At near confluence, HepG2 cells were treated with lysine, proline and ascorbate (400, 140, and 100 µg/mL, respectively) with variable amounts of EGCG (0, 10, 20 or 50 µg/mL) dissolved in media. In subsequent studies, HepG2 and SK-Hep-1 cells were treated with the nutrient mixture, dissolved in media and tested at 0, 10, 50, 100, 500, and 1,000 µg/mL in triplicate at each dose. Parallel sets of cultures were treated with PMA (100 ng/mL) for induction of MMP-9. Control and PMA treatments were done in triplicates. The plates were then returned to the incubator. The conditioned media were collected separately, pooled, and centrifuged at 4 °C for 10 min at 3,000 rpm to remove cells and cell debris. The supernatant was collected and used to assess for u-PA activity (by fibrin zymography on 10% SDS-PAGE gels containing fibrinogen and plasminogen), MMP-2 and -9 (by gelatinase zymography), and TIMPs (by reverse zymography).

### 3.4. MTT Assay

Cell viability was evaluated by MTT assay, a colorimetric assay based on the ability of viable cells to reduce a soluble yellow tetrazolium salt [3-(4,5-dimethylthiazol-2-yl)-2,5-diphenyltetrazolium bromide] (MTT) to a blue formazan crystal by mitochondrial succinate dehydrogenase activity of viable cells. This test is a good index of mitochondrial activity and thus of cell viability. After 24 h incubation, the cells were washed with phosphate buffered saline (PBS) and 500 μL of MTT (Sigma #M-2128) 0.5 mg/mL in media was added to each well. After MTT addition (0.5 mg/mL) the plates were covered and returned to the 37 °C incubator for 2 h, the optimal time for formazan product formation. Following incubation, the supernatant was carefully removed from the wells, the formazan product was dissolved in 1 mL DMSO, and absorbance was measured at 570 nm in Bio Spec 1601, Shimadzu spectrometer. The OD_570_ of the DMSO solution in each well was considered to be proportional to the number of cells. The OD_570_ of the control (treatment without supplement) was considered 100%.

### 3.5. Fibrin Zymography

Fibrin zymography was used to analyze u-PA activity on 10% SDS-PAGE gels containing fibrinogen (5.5 mg/mL) and plasminogen (50 μg/mL). After electrophoresis, the gels were washed twice with 2.5% Triton X-100 for 30 min. The gels were then incubated overnight at 37 °C with 0.1% glycine buffer pH 7.5 and then stained with 0.5% Coomassie Brilliant Blue R250 and destained. Electrophoresis of u-PA was conducted for comparison. Fibrin zymograms were scanned using CanoScan 9950F Canon Scanner.

### 3.6. Gelatinase Zymography

Gelatinase zymography was performed in 10% NOVEX Pre-Cast SDS Polyacrylamide Gel (Invitrogen Corporation, Carlsbad, CA, USA) in the presence of 0.1% gelatin under non-reducing conditions. Culture media (20 μL) were mixed with sample buffer and loaded for SDS-PAGE with tris glycine SDS buffer as suggested by the manufacturer (Novex). Samples were not boiled before electrophoresis. Following electrophoresis the gels were washed twice in 2.5% Triton X-100 for 30 min at room temperature to remove SDS. The gels were then incubated at 37 °C overnight in substrate buffer containing 50mM Tris-HCl and 10 mM CaCl_2_ at pH 8.0 and stained with 0.5% Coomassie Blue R250 in 50% methanol and 10% glacial acetic acid for 30 min and destained. Upon renaturation of the enzyme, the gelatinases digest the gelatin in the gel and give clear bands against an intensely stained background. Protein standards were run concurrently and approximate molecular weights were determined by plotting the relative mobilities of known proteins.

### 3.7. Reverse Zymography

TIMPS were analyzed by reverse zymography on 15% SDS gels containing serum-free conditioned medium from cells. After electrophoresis the gels were washed twice with 2.5% Triton-X for 30 min at room temperature to remove SDS. The gels were then incubated at 37 °C overnight in 50 mM Tris-HCl and 10 mM Ca Cl_2_ at pH 7.6 and stained with 0.5% Coomassie Blue R25, destained and scanned.

### 3.8. Scanning of Gelatinase and Fibrin Zymograms

Gelatinase and fibrin zymograms were scanned using CanoScan 9950F Canon scanner at 300 dpi. The intensity of the bands was evaluated using the pixel-based densitometer program Un-Scan-It, Version 5.1, 32-bit, by Silk Scientific Corporation (Orem, UT, USA), at a resolution of 1 Scanner Unit (1/100 of an inch for an image that was scanned at 100 dpi). The pixel densitometer calculates the optical density of each pixel (values 0 to 255) using the darkly stained background of the gel as a pixel value of 0. A logarithmic optical density scale was used since the optical density of films and gels is logarithmically proportional to the concentration. The pixel densitometer sums the optical density of each pixel to give a band’s density. In all graphs, band densities were reported as percentages of the sums of all pixels in a given lane (treatment) of a gel.

### 3.9. Matrigel Invasion

Invasion studies were conducted using Matrigel (Becton Dickinson, San Jose, CA, USA) inserts in 24-well plates. Suspended in medium, HepG2 cells were supplemented with nutrients, as specified in the design of the experiment and seeded on the insert in the well. Thus both the medium on the insert and in the well contained the same supplements. The plates with the inserts were then incubated in a culture incubator equilibrated with 95% air and 5% CO_2_ for 24 h. After incubation, the media from the wells were withdrawn. The cells on the upper surface of the inserts were gently scrubbed away with cotton swabs. The cells that had penetrated the Matrigel membrane and migrated onto the lower surface of the Matrigel were stained with hematoxylin and eosin and visually counted under the microscope.

### 3.10. Apoptosis

At near confluence, HepG2 cells were challenged with NM dissolved in media at 0, 100, 500, and 1,000 µg/mL and incubated for 24 h. The cell culture was washed with PBS and treated with the caspase reagent as specified in the manufacturer’s protocol (Molecular Probes Image-IT™ Live Green Poly Caspases Detection Kit 135104, Invitrogen). The cells were photographed under a fluorescence microscope and counted. Green-colored cells represent viable cells, while yellow orange represents early apoptosis and red late apoptosis.

### 3.11. Statistical Analysis

The results were expressed as means ± SD, as indicated in the results, for the groups. Data was analyzed by independent sample “t” test using MedCalc Software (Markakerke, Belgium).

## 4. Conclusions

Our study demonstrated that the synergistic activity of the nutrient mixture was significantly more potent in inhibiting hepatocellular HepG2 cell growth, MMP expression and invasion through Matrigel than the activity of individual components of NM at equivalent doses, such as EGCG alone or the combination of EGCG and LPA. The superior potency of NM over EGCG + LPA and of EGCG + LPA over EGCG alone can be understood from the more comprehensive treatment offered by the combination of nutrients in NM over individual components of NM since cellular growth, secretion of MMPs and invasion are mediated by complex biochemical pathways. In addition, NM demonstrated significant dose-dependent induction of HepG2 cell apoptosis.

Our previous *in vivo* studies demonstrated the potency of NM in inhibition of SK-Hep-1 xenograft tumor growth and hepatic metastasis of B16FO melanoma cells. Our current *in vitro* investigation of the efficacy of NM on modulation of SK-Hep-1 cell secretion of MMPs, u-PA and TIMPs, critical to tumor invasion and metastasis, supported these previous findings. NM significantly inhibited SK-Hep-1 u-PA and MMP-2 and -9 secretion and up-regulated secretion of TIMPs.

In conclusion, the NM demonstrated significant synergistic antitumor effects on hepatocellular carcinoma *in vivo* and *in vitro*, suggesting NM has therapeutic potential in treatment of HCC.
